# Use of ultrasound in the assessment of screen-detected malignant microcalcifications

**DOI:** 10.1186/bcr3295

**Published:** 2012-11-09

**Authors:** MZ Mvere, EJ Cornford, JJ James

**Affiliations:** 1 Nottingham Breast Institute, Nottingham, UK

## Introduction

The aim of this study was to assess radiological and pathological features associated with successful ultrasound-guided biopsy of malignant microcalcifications.

## Methods

Screen-detected breast cancers were reviewed. Cases where microcalcification was the predominant mammographic feature were identified. Ultrasound findings, mammographic appearances, method of biopsy and pathological features were recorded.

## Results

There were 348 breast cancers diagnosed in the 24-month study period, 84 cases had microcalcification as the predominant mammographic feature. In 75 cases (89%) an ultrasound scan was performed. Forty-one cases had an ultrasound abnormality and underwent an ultrasound-guided core biopsy. Successful ultrasound-guided core biopsy of malignant microcalcifications was associated with a mammographic size >20 mm (*P *= 0.002), the presence of an associated mammographic feature (*P *= 0.03) and invasive disease on surgical resection (*P *= 0.009). There was no association with calcification morphology, mammographic background pattern or the histological grade of the DCIS. When the ultrasound was normal or the ultrasound-guided core biopsy inconclusive, the patient underwent an additional biopsy typically stereotactic vacuum biopsy. In the 41 cases where ultrasound-guided core was the first biopsy performed, nine cases underwent an additional biopsy prior to the therapeutic surgical procedure, compared with just one case when stereotactic vacuum biopsy was the first procedure (*P *= 0.005).

## Conclusion

Ultrasound-guided core biopsy of malignant microcalcifications is more likely to be successful when mammographic size is larger and invasive disease is present. When ultrasound-guided biopsy is the first-line procedure a second biopsy is significantly more likely prior to therapeutic surgery.

